# Plasma ammonia concentrations in extremely low birthweight infants in the first week after birth: secondary analysis from the ProVIDe randomized clinical trial

**DOI:** 10.1038/s41390-019-0730-z

**Published:** 2020-01-02

**Authors:** Barbara E. Cormack, Yannan Jiang, Jane E. Harding, Caroline A. Crowther, Adrienne Lynn, Arun Nair, Michael Hewson, Mike Meyer, Roland Broadbent, Dianne Webster, Emma Glamuzina, Bryony Ryder, Frank H. Bloomfield

**Affiliations:** 1 Liggins Institute, University of Auckland, Auckland, New Zealand; 2 Newborn Services, Auckland City Hospital, Auckland, New Zealand; 3 Neonatal Intensive Care Unit, Christchurch Women’s Hospital, Christchurch, New Zealand; 4 Newborn Intensive Care Unit, Waikato Hospital, Hamilton, New Zealand; 5 Neonatal Intensive Care Unit, Wellington Hospital, Wellington, New Zealand; 6 Neonatal Unit, Middlemore Hospital, Auckland, New Zealand; 7 Neonatal Intensive Care Unit, Dunedin Hospital, Dunedin, New Zealand; 8 LabPlus, Auckland District Health Board, Auckland, New Zealand; 9 National Adult and Paediatric Metabolic Service, Auckland, New Zealand

## Abstract

**Background:**

Little is known about normative ammonia concentrations in extremely low birthweight (ELBW) babies and whether these vary with birth characteristics. We aimed to determine ammonia concentrations in ELBW babies in the first week after birth and relationships with neonatal characteristics and protein intake.

**Methods:**

Arterial blood samples for the measurement of plasma ammonia concentration were collected within 7 days of birth from ProVIDe trial participants in six New Zealand neonatal intensive care units.

**Results:**

Three hundred and twenty-two babies were included. Median (range) gestational age was 25.7 (22.7–31.6) weeks. Median (interquartile range (IQR)) ammonia concentration was 102 (80–131) µg/dL. There were no statistically significant associations between ammonia concentrations and birthweight or sex. Ammonia concentrations were weakly correlated with mean total (Spearman’s *r*_s_ = 0.11, *P* = 0.047) and intravenous (*r*_s_ = 0.13, *P* = 0.02) protein intake from birth, gestational age at birth (*r*_s_ = −0.13, *P* = 0.02) and postnatal age (*r*_s_ = −0.13, *P* = 0.02).

**Conclusions:**

Plasma ammonia concentrations in ELBW babies are similar to those of larger and more mature babies and only weakly correlated with protein intake. Currently, recommended thresholds for investigation of hyperammonaemia are appropriate for ELBW babies. Protein intake should not be limited by concerns about potential hyperammonaemia.

## Introduction

Ammonia is a normal constituent of body fluids, but at high concentrations it is a known neurotoxin.^1^ A high plasma ammonia concentration indicates nitrogen homeostasis dysfunction caused either by increased ammonia production exceeding the body’s capacity to eliminate ammonia or by decreased elimination, as it may occur with porto-systemic shunts or inborn errors of nitrogen metabolism, such as urea cycle defects. In the first week after birth, disorders of nitrogen metabolism and transient hyperammonaemia can result in severe hyperammonaemia in the range of 852–5109 µg/dL (to convert ammonia to µmol/L, multiply values by 0.5872).^2,3^ Hyperammonaemia can also be caused by intravenous fibrin or casein hydrolysates, arginine-deficient intravenous nutrition or by starvation causing massive protein catabolism.^4–6^ In newborns, it typically presents with poor feeding, vomiting, lethargy, respiratory alkalosis and irritability that rapidly progresses to seizures and coma.^7^ Irreversible damage to the developing brain may occur, including cortical atrophy, ventricular enlargement, demyelination and grey and white matter hypodensities.^1,8–11^ The extent of the damage depends on the stage of brain maturation and the magnitude and duration of ammonia exposure.^10,12^ If left untreated, hyperammonaemia can be fatal.^9^ Ammonia concentrations exceeding 341–852 µg/dL are of concern^13^ as 50% of patients with plasma ammonia concentrations over 341 µg/dL have inborn errors of metabolism,^14^ but evidence-based criteria to diagnose hyperammonaemia in extremely low birthweight (ELBW) babies are lacking.

Reference intervals to interpret plasma ammonia concentrations are crucial for informed clinical decision making. Published reference ranges for plasma ammonia concentrations of healthy babies within 30 days of birth are 36–162 µg/dL and decrease with age up to 14 years.^8^ For preterm neonates, investigation of possible inherited metabolic disorders is recommended when the plasma ammonia concentration is >256 µg/dL, the concentration at which mild symptoms such as vomiting, lethargy and confusion develop.^8^ However, substantially higher ammonia concentrations previously have been reported in small groups of mainly moderate- to late-preterm babies without metabolic disorders.^3,4,6,15^ Furthermore, little is known about normal ammonia concentrations in ELBW (<1000 g) babies or whether ammonia concentrations vary with gestational age, sex, birthweight of the baby, smallness for gestational age (SGA), postnatal age or protein intake.^16^

Up to 50% of ELBW babies have some neurodevelopmental impairment in childhood.^17^ Current consensus preterm intravenous nutrition recommendations are that protein (amino acid) supply should begin on the first postnatal day with at least 1.5 g/kg per day to achieve an anabolic state and increase to 2.5 to 3.5 g/kg per day on day 2.^18^ Others recommend higher amino acid intakes to achieve fetal growth, optimal neurodevelopment and long-term health.^19^ However, there is concern about the ability of ELBW babies to metabolize specific amino acids,^20^ with the potential consequence that high intravenous amino acid intakes may increase ammonia to concentrations that are toxic to the developing brain contributing to less optimal neurodevelopmental outcomes.^21^ In some institutions, this limits use of amino acids from birth.^22^ However, as ammonia concentrations are not routinely measured in neonatal care or research, it is not known to what extent the quantum and type of intravenous amino acid solution increase ammonia concentrations in ELBW babies. A critical gap therefore exists in accurate and up-to-date reference values for ELBW babies.

Our aim was to determine whether plasma ammonia concentrations in ELBW babies in the first week after birth differ from those previously published for moderately preterm and term neonates and specifically to investigate:

1. The relationships between plasma ammonia concentrations and gestational age at birth, birthweight, sex of the baby, SGA, postnatal age and protein intake.
2. Associations between early plasma ammonia concentrations and clinical outcomes assessed at 36 weeks corrected gestational age.

## Methods

This cohort comprised a subgroup of ELBW babies who participated in the ProVIDe trial (Australian New Zealand Clinical Trials Registry: ACTRN12612001084875 https://anzctr.org.au/Trial/Registration/TrialReview.aspx?id = 363124), a multicentre, two-arm, double-blind, parallel, randomized, controlled trial. The Northern B Health and Disability Ethics Committee gave ethical approval for the study (No. 13/NTB/84), and each participating site had institutional approval through local institutional review processes. Informed written consent was obtained for all participants from their parents or caregivers.

The ProVIDe study protocol has been published elsewhere.^23^ Briefly, in addition to standard nutritional support according to each participating unit’s policies, 434 ELBW participants were randomized at 1:1 ratio between April 2014 and October 2018 to receive either 1 g per day of protein as amino acid solution (TrophAmine®, B Braun Medical, Irvine, CA), or placebo (saline) administered through the umbilical arterial catheter for the first 5 days after birth. Inclusion criteria were birthweight <1000 g and placement of an umbilical arterial catheter. Exclusion criteria were: admission to neonatal intensive care more than 24 h after birth; multiple births of more than two babies; known chromosomal or genetic abnormality; congenital disorder affecting growth; inborn error of metabolism, and danger of imminent death.

The primary outcome of the trial is survival free from neurodevelopmental disability at 2 years corrected age, expected to be available in 2021. Baseline nutritional intakes were not mandated, meaning that participants in both the intervention and placebo groups received a range of protein intakes due to the differences in unit nutrition policies and the clinical decisions made for each baby, and received one of two intravenous amino acid solutions: TrophAmine®, B Braun Medical (two sites), or Primène® Baxter Healthcare Ltd, Sydney, Australia (four sites), as their standard intravenous nutrition. Protein intakes in both arms of the study were within current internationally reported ranges in observational studies.^24–30^ The subgroup reported here comprises participants admitted to the six recruiting centres within New Zealand (NZ). Nutritional intake data were collected prospectively and used to estimate mean daily intravenous, enteral and total protein intakes^31^ from birth until the end of the day the blood sample was collected, and also the mean intake on the day of sample collection. As the primary outcome for the randomized trial is at 2 years corrected age and is not complete, this analysis was conducted as a cohort without unblinding group allocation.

The data were extracted by a third party and recoded with dummy IDs to avoid unblinding study investigators. Thus, actual amino acid intakes for all babies were used in the analyses, including the additional amino acids administered through the umbilical arterial catheter in babies randomized to the intervention arm. However, the analysis of ammonia concentrations according to brand of amino acid solution administered was carried out using data from participants randomized to the placebo arm only.

An arterial blood sample for the measurement of plasma ammonia concentration was collected after the 5-day intervention ceased. If the intervention ceased earlier than day 5, the sample was taken on the day the intervention ceased. The blood sample (0.5 mL) was drawn from an indwelling umbilical artery catheter and placed in a paediatric EDTA tube, covered with ice, and immediately transported to the site laboratory. Ammonia concentrations were determined only on samples that met the local laboratory analytical criteria for collection, receipt and preparation of samples. Analysis was by quantitative enzymatic method on a Roche/Hitachi Cobas 311/501, Abbott Cobas 8000 or Roche Cobas 8000 analyser. Ammonia concentrations >256 µg/dL were reported to the ProVIDe trial data monitoring committee and neonatologist responsible for clinical care of the baby by the site investigator.

### Statistical analysis

All participants’ data were stored in a secure study database and imported to SAS version 9.4 (SAS Institute Inc., Cary, NC) for analysis. Statistical tests were two-sided at a 5% significance level. Descriptive summaries are presented using median (range) for continuous variables, and number (%) for categorical variables. Correlations between ammonia concentrations and gestational age at birth, birthweight, postnatal age at the time of sampling and protein intake were tested using Spearman’s correlation coefficients with associated *p* values. Differences in ammonia concentrations between boys and girls, SGA babies (defined as birthweight <10th percentile^32^) and those appropriate for gestational age (AGA), and for TrophAmine and Primène groups were tested using Wilcoxon’s two-sample tests.

The association between ammonia concentration and the following clinical outcomes (assessed at 36 weeks corrected gestational age) were also investigated: intraventricular haemorrhage (IVH), defined as severe IVH (grade 3 or 4 defined using the grading system from Papile et al. ^33^) chronic lung disease (need for oxygen at 36 weeks post-menstrual age or 28 days after birth if born after 32 weeks gestation); retinopathy of prematurity grades as per the International Classification of Retinopathy of Prematurity;^34^ necrotizing enterocolitis defined as Bell’s stage 2 or higher;^35^ patent ductus arteriosus (PDA) diagnosed by echocardiography needing treatment; early-onset sepsis (EOS: birth to day 7) and late-onset sepsis (LOS: beyond 7 days after birth) defined as a positive bacterial culture in cerebrospinal fluid, urine or blood with clinical signs of infection and with antibiotics for 5 or more days with the intention of treating an infection, or treatment for a shorter period if the patient died; and death prior to discharge from neonatal care. In addition, we categorized babies who did not have any of the above clinical outcomes as ‘well’ babies and compared ammonia concentrations in this group with the remaining babies who had one or more of the above outcomes (‘unwell’). All clinical outcomes were analysed as a binary indicator of Yes or No, using multiple logistic regression models with a logit link controlling for gestational age at birth, sex and postnatal age at the time of sampling. Adjusted odds ratios (ORs), 95% confidence interval (CI), and *p* value are reported.

## Results

**Fig. 1:**
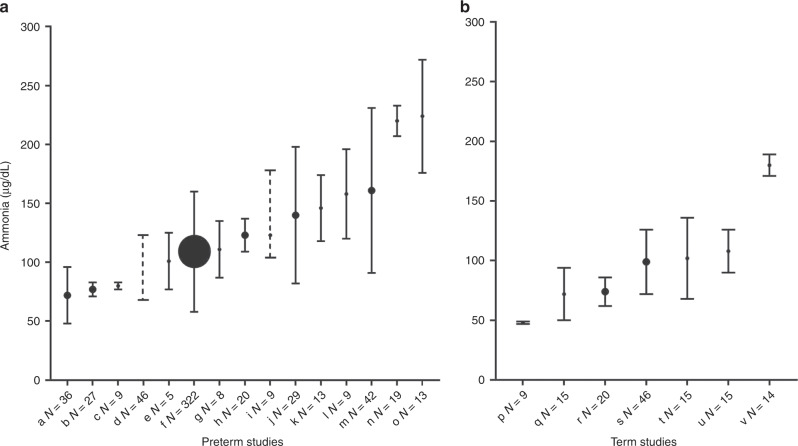
Published ammonia concentrations in newborn babies. Plasma ammonia concentrations reported in previous publications and in our study (*N* = 322) for preterm (**a**) and term-born (**b**) babies 1958–2008, in order of lowest to highest mean concentrations. The *x*-axis label (a–v) indicates first author (below) and *N* = sample size. Preterm: a Usmani^35^, b Batshaw (SGA)^46^, c Batshaw (AGA)^46^, d Batshaw (1986)^41^, e Rivera (amino acids + glucose)^39^, f our study, **g** Rivera (glucose alone)^39^, h Yalcin^43^, i Sanchez^15^, j Shohat^38^, k Thomas (Travasol)^6^, l Blanco^40^, m Beddis^3^, n Seashore^34^, o Thomas^6^ (FreAmine II). Term: p Batshaw (AGA)^46^, q Rubaltelli (SGA)^30^, r Yalcin^43^, s Clemmens^45^, t Oberholzer^46^, u Rubaltelli (AGA)^30^, v Seashore^34^.  show mean (dot) and whiskers show standard deviation, show mean and range and  show range only. Dot size indicates size of the study,  <20 participants,  20–50 participants,  our study.

Of the 434 babies randomized in the ProVIDe study, 382 (88%) were recruited in NZ hospitals. Plasma ammonia concentrations were available for 324 (85% of NZ babies). Two participants with plasma ammonia concentrations of >1400 µg/dL were considered to be in the range of metabolic pathology and were excluded.^14^ The first was being investigated for an inborn error of metabolism when they died. No cause was found in the second case and the baby died 6 weeks later. This left samples from 322 babies for analysis. The median (range) gestation at birth was 25.7 (22.7–31.6) weeks, birthweight 783 (450–998) g, age at blood sampling 5 (2–7) days. Thirty-seven babies (12%) were SGA and 147 (46%) were boys. Most blood samples (72%) were taken on day 5 (Table 1). More babies who did not have a sample for ammonia concentration died (27.6% vs. 16.1%, *p* = 0.02), but there was no difference in gestational age, SGA status or sex between babies who did not have an ammonia sample compared with those who did. The median (IQR; 95th percentile) ammonia concentration was 102 (80–131; 187) µg/dL. This is similar to the previously reported values in Fig. 1. Four participants (1%) had ammonia concentrations >256 µg/dL.

**Table 1:** Characteristics and protein intake of the study cohort.

Characteristic	*n* (%)
Postnatal age at sampling (day)	
1	4 (1)
2	16 (5)
3	34 (11)
4	28 (9)
5	233 (72)
6	6 (2)
7	1 (<1)
Died before discharge	51 (16)
Male	147 (46)
Small for gestational age	37 (12)
Clinical outcome	
Necrotizing enterocolitis	40 (12)
Early-onset sepsis	7 (2)
Late-onset sepsis	110 (34)
Patent ductus arteriosus	140 (44)
Intraventricular haemorrhage	
None	223 (69)
Grade 1	45 (14)
Grade 2	20 (6)
Grade 3	8 (3)
Grade 4	21 (7)
Grade 5	3 (1)
Chronic lung disease	208 (65)
Retinopathy of prematurity	
None	116 (36)
Stage 1	66 (21)
Stage 2	64 (20)
Stage 3	38 (12)
Stage 4	1 (<1)
No eye exam performed	35 (11)
Missing	2 (<1)
Mean daily protein intake (g/kg per day)	Mean (SD)
On sample day	
Intravenous	3.41 (1.08)
Enteral	0.29 (0.33)
Total	3.70 (1.07)
From birth to sample day	
Intravenous	3.10 (0.81)
Enteral	0.14 (0.13)
Total	3.24 (0.82)

### Associations with GA, birthweight, time of sampling, SGA and sex

**Fig. 2:**
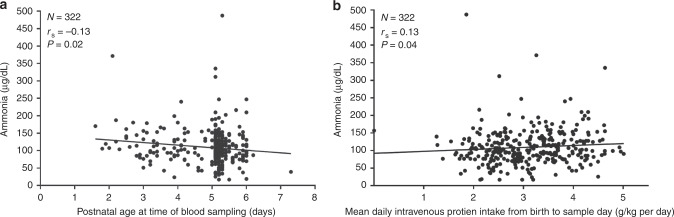
Plasma ammonia concentrations in the first week after birth. Correlation between plasma ammonia concentration and postnatal age at the time of blood sampling (**a**) and mean daily intravenous protein intake from birth to sample day (**b**).

Weak negative correlations were observed between ammonia concentration and gestational age at birth (Spearman’s *r*_s_ = −0.13, *P* = 0.022), and postnatal age (*r*_s_ = −0.13, *P* = 0.018) (Fig. 2a), but there was no association between ammonia concentration and birthweight (*r*_s_ = 0.002, *P* = 0.97). The median (IQR) ammonia concentration was similar in girls and boys (102 (83–136) µg/dL vs. 101 (77–128) µg/dL, *P* = 0.28) and in SGA and AGA babies (104 (75–119) µg/dL vs. 101 (82–131) µg/dL, *P* = 0.67).

### Associations with protein intake and amino acid source

**Fig. 3:**
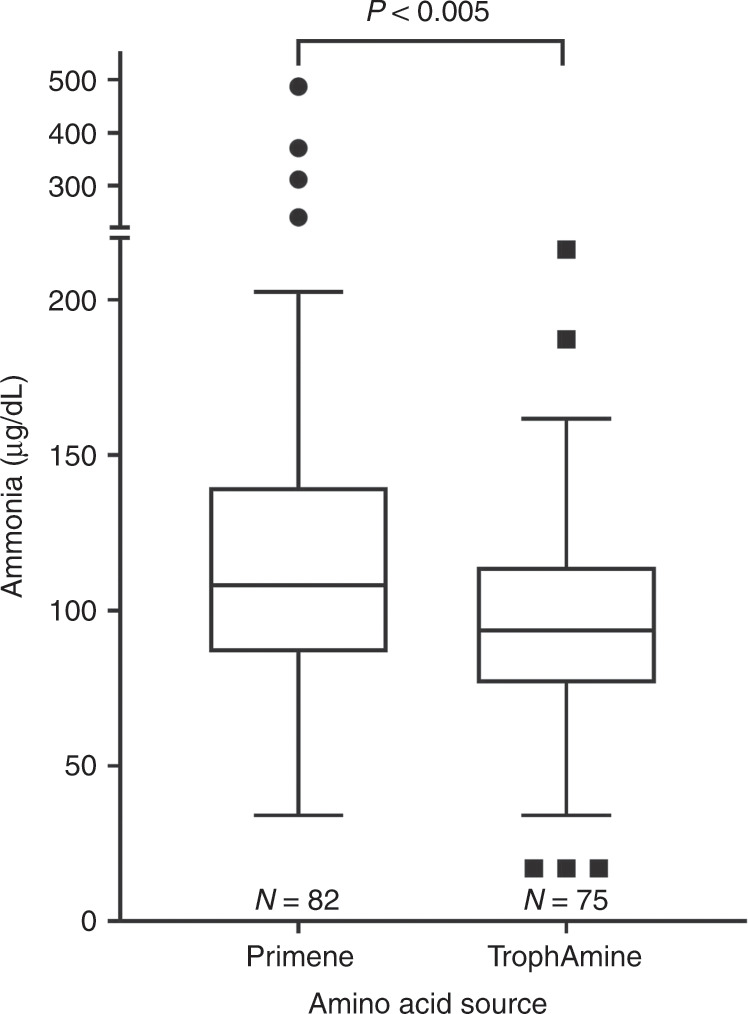
Comparison of plasma ammonia concentration between the two amino acid brands (Wilcoxon’s two-sample test). Data are represented as Tukey’s boxplots, with the bottom and the top of the box representing the first and third quartiles; the band inside the box, the median; the whiskers, 1.5× the interquartile range; and the circles, outliers.

Total protein intake on the sample day and mean total protein intake from birth to sample day were means (SD) 3.70 (1.07) and 3.24 (0.82) g/kg per day, with only a small contribution from enteral protein intake (Table 1). There was no significant correlation between ammonia concentration and intravenous (*r*_s_ = 0.11, *P* = 0.06), enteral (*r*_s_ = −0.08, *P* = 0.16) or total protein (*r*_s_ = 0.08, *P* = 0.14) intakes on the day of blood sample collection. There was a weak correlation between ammonia concentration and both intravenous (*r*_s_ = 0.13, *P* = 0.02, Fig. 2b) and total (*r*_s_ = 0.11, *P* = 0.047), but not enteral (*r*_s_ = −0.07, *P* = 0.22), mean daily protein intakes from birth to sample day. The median ammonia concentration was 16% higher when Primène was the amino acid source (Fig. 3).

### Associations with clinical outcomes

Babies with a PDA had slightly higher median (IQR) ammonia concentrations (109 (85–139) µg/dL) than those babies with no PDA (99 (77–128) µg/dL, *P* = 0.01). After adjustment for gestational age at birth, sex and postnatal age at the time of plasma sampling, there was a statistically, although not clinically, significant association between plasma ammonia concentrations and PDA (adjusted OR 1.10, 95% CI [1.01, 1.22], *P* = 0.03, that is, for every 10 µg/dL increase in ammonia concentration, the odds of PDA increase by 10%). No significant association was found between ammonia concentration and any other clinical outcomes at 36 weeks corrected gestational age. The median (IQR) plasma ammonia concentration of those who were categorized as unwell (*n* = 286, 84 (63–109) µg/dL) was similar to those who were well (*n* = 38, 83 (70–104) µg/dL, *P* = 0.87). Fifty-one (16%) of the 322 babies died before discharge from neonatal intensive care. The median (IQR) plasma ammonia concentration of those who died (111 (92–150) µg/dL) was higher than those who survived (101 (77–128) µg/dL, *P* = 0.03). However, the association was no longer statistically significant after adjustment (adjusted OR 1.01; 95% CI [0.99, 1.02], *P* = 0.09).

## Discussion

We found that ammonia concentrations in ELBW babies are similar to those of the predominantly larger preterm and term babies previously reported and summarized in Fig. 1. There was no effect of birthweight on ammonia concentration in our ELBW cohort, and the effect of protein intakes within, or even above, currently recommended ranges on plasma ammonia concentrations is minimal. Indeed, the weakly negative correlation between ammonia concentration and postnatal age supports the finding of minimal or no effect of protein intake, because current standard neonatal nutrition practice at all the sites is to gradually increase protein intake from days 2 to 5 after birth.

To our knowledge, this is the largest published dataset of neonatal plasma ammonia concentrations for ELBW babies. Previous studies over the past 60 years have had small sample sizes and reported wide variation in ammonia concentrations (Fig. 1). This is likely due to differences in procedures for collecting and preserving specimens, types of laboratory tests and in the blood sampled, that is, venous, arterial or capillary.^36^ The measurement of plasma ammonia concentration is greatly influenced by both pre-analytical and analytic delays after collection, which may lead to falsely high results.^37,38^ In particular, ammonia concentration increases spontaneously in blood after collection and is stable for <15 min at 4 °C.^39^

The previous studies of ammonia concentrations shown in Fig. 1 mostly predate modern neonatal intensive care. One of the few studies of ELBW babies reported ammonia concentrations >153 µg/dL in five of nine ventilated babies and higher maximum ammonia concentrations compared with our findings, but this was in the early era (1978) of neonatal intensive care.^15^ Other studies of preterm and low birthweight babies from this era reported ammonia concentrations >170 and as high as 313 µg/dL.^36,40,41^ Prior to the introduction of crystalline amino acid solutions, the fibrin or casein hydrolysates used as the nitrogen source for intravenous nutrition solutions contained significant amounts of preformed ammonia, which may, at least partially, explain the higher ammonia concentrations in these early studies.^4–6,42^ Subsequent changes in nutritional management, such as the use of expressed breastmilk and whey-predominant rather than casein-predominant infant formula, the quantity of protein in enteral feeds,^43^ amount and timing of intravenous amino acid administration,^6,40,44^ and a more aggressive approach to initiation and advancement of enteral feeding may also have contributed to lower ammonia concentrations in our cohort compared with previously reported results.^45^ However, ammonia concentrations >153 µg/dL also have been reported more recently. In a 2008 study of 61 babies, Blanco et al.^21^ reported nine babies with a blood urea nitrogen >60 mg/dL had peak ammonia concentrations in the first week ranging from 165 to 210 µg/dL with intravenous protein intakes of 2.5 to 4 g/kg per day.^21^ In our study, a few ammonia concentrations were also in this range but the consequences of this, if any, are currently unknown.^46^ The significantly lower ammonia concentrations in the TrophAmine cohort compared with the Primène cohort may be due to differences in amino acid profiles between these two solutions. For instance, arginine, deficiency of which has previously been associated with hyperammonaemia,^5,47^ is 43% higher in TrophAmine than Primène.

In our cohort, both gestational age at birth and postnatal age had a small negative effect on plasma ammonia concentration. One other small study in moderate- to late-preterm babies found a negative correlation between ammonia concentration and gestational age at birth.^48^ Others have found no effect of gestational age in very low birthweight babies.^41^ In contrast to our cohort, a negative correlation with postnatal age has previously been reported in both preterm^41^ and term babies.^49^

As previously reported by others in term babies,^50^ we found no effect of birthweight or sex on ammonia concentrations. In our study, mean ammonia concentrations of SGA and AGA babies were similar as found by others in low birthweight babies.^51^ One small study has reported lower ammonia concentrations in SGA babies.^48^ Inconsistency in these results is likely due to the small sample sizes in previous studies.

It has been previously suggested that there may be an association between some clinical outcomes and plasma ammonia concentrations. A high plasma ammonia concentration has been shown to increase intracranial pressure in rhesus monkeys, and both anoxia and shock have been shown to elevate the ammonia concentration in isolated nervous tissue in animals.^52^ Hyperammonaemia in babies has been reported after perinatal asphyxia^53^ and therefore hypoxia due to respiratory distress syndrome might contribute to elevated plasma ammonia concentrations. We found no correlation between any of these clinical outcomes and plasma ammonia concentrations in this large of cohort of ELBW babies. However, babies with a PDA had significantly higher unadjusted and adjusted ammonia concentrations, although the effect size is small. To our knowledge, this has not been reported by others and might be explained by a patent ductus venosus in addition to a PDA. Our finding in unadjusted, but not adjusted, analyses that babies who died prior to discharge had higher ammonia concentrations in the first week after birth than those who survived to discharge needs verification in further studies.

## Limitations

A potential weakness is that samples were analysed at different sites and taken over the first 5 days after birth, although the majority were on day 5.

## Conclusion

This is a unique dataset of normal plasma ammonia concentrations in a cohort of ELBW babies receiving a range of protein intakes up to 5 g/kg per day. The sample size is large in comparison with previous studies and the participants originate from six different NZ sites with laboratories using the same method of ammonia analysis. Hence, plasma ammonia concentrations are generalizable to ELBW populations outside NZ. We conclude that currently recommended thresholds for investigation of hyperammonaemia are appropriate for ELBW babies and concerns that protein intakes at the upper end of recommended ranges lead to hyperammonaemia are unfounded.

## Supplementary information

CONSORT Checklist –Ammonia
