# Supplementary material for: Plasma ammonia concentrations in extremely low birthweight infants in the first week after birth: secondary analysis from the ProVIDe randomized clinical trial
Source: Pediatr Res. 2020 Jan 2;88(2):250–6. doi: 10.1038/s41390-019-0730-z (PMC7384986; doi:10.1038/s41390-019-0730-z)
Supplement: Supplementary file 1 — CONSORT Checklist –Ammonia [file 41390_2019_730_MOESM1_ESM.docx]

CONSORT CHECKLIST

#

Item

Reported on

Section and Topic Title and abstract

Introduction

Background

and objectives

Methods

No. Checklist Item

1a Identification as a randomized trial in the title

1b Structured summary of trial design, methods, results, and conclusions (for specific guidance see CONSORT for abstracts)

2a Scientific background and explanation of rationale 2b Specific objectives or hypotheses

Page No.

# N/A

1-2

3-4

5

Trial design 3a Description of trial design (such as parallel, factorial) including allocation ratio

3b Important changes to methods after trial commencement (such as eligibility criteria), with reasons Participants 4a Eligibility criteria for participants

4b Settings and locations where the data were collected

Interventions 5 The interventions for each group with sufficient details to allow replication, including how and when they were actually administered

Outcomes 6a Completely defined prespecified primary and secondary outcome measures, including how and when they were assessed

6b Any changes to trial outcomes after the trial commenced, with reasons

Sample size 7a How sample size was determined

7b When applicable, explanation of any interim analyses and stopping guidelines

# 6

N/A

6

6

6-8

5

N/A

6-7

N/A

Randomization Sequence

generation

Allocation concealment mechanism

8a Method used to generate the random allocation sequence

8b Type of randomization; details of any restriction (such as blocking and block size)

9 Mechanism used to implement the random allocation sequence (such as sequentially numbered containers), describing any steps taken to conceal the sequence until interventions were assigned

# Ref 23

6

Ref 23

Implementation 10 Who generated the random allocation sequence, who enrolled participants, and who assigned participants to interventions

Blinding 11a If done, who was blinded after assignment to interventions (for example, participants, care providers, those assessing outcomes) and how

11b If relevant, description of the similarity of interventions

# Ref 23

6-7

N/A

Statistical

methods

Results

Participant flow

(a diagram is strongly recommended)

12a Statistical methods used to compare groups for primary and secondary outcomes 12b Methods for additional analyses, such as subgroup analyses and adjusted analyses

13a For each group, the numbers of participants who were randomly assigned, received intended treatment, and were analyzed for the primary outcome

13b For each group, losses and exclusions after randomization, together with reasons

# 8

8-9

9

9

Recruitment 14a Dates defining the periods of recruitment and follow-up

14b Why the trial ended or was stopped

Baseline data 15 A table showing baseline demographic and clinical characteristics for each group

Numbers analyzed 16 For each group, number of participants (denominator) included in each analysis and whether the analysis was by original assigned groups

# 7

Ref 23

Ta ble 1

9-10

Outcomes

and estimation

17a For each primary and secondary outcome, results for each group, and the estimated effect size and its precision (such as 95% confidence interval)

17b For binary outcomes, presentation of both absolute and relative effect sizes is recommended

# 9-10

9-10

Ancillary analyses 18 Results of any other analyses performed, including subgroup analyses and adjusted analyses, distinguishing prespecified from exploratory

Harms 19 All important harms or unintended effects in each group (for specific guidance see CONSORT for harms)

Comment

Limitations 20 Trial limitations, addressing sources of potential bias, imprecision, and, if relevant, multiplicity of analyses Generalizability 21 Generalizability (external validity, applicability) of the trial findings

# 9-10

9

14

14-15

Interpretation 22 Interpretation consistent with results, balancing benefits and harms, and considering other relevant evidence 12-14

Other information

Registration 23 Registration number and name of trial registry

Protocol 24 Where the full trial protocol can be accessed, if available

Funding 25 Sources of funding and other support (such as supply of drugs), role of funders

# 6

6

17

a We strongly recommend reading this statement in conjunction with the CONSORT 2010 Explanation and Elaboration for important clarifications on all the items. If relevant, we also recommend reading CONSORT extensions for cluster randomized trials, noninferiority and equivalence trials, nonpharmacological treatments, herbal interventions, and pragmatic trials. Additional extensions are forthcoming: for those and for up-to-date references relevant to this checklist, see [http://www.consort-statement.org.](http://www.consort-statement.org/)
